# Immune Suppressive Extracellular Vesicle Proteins of *Leptopilina heterotoma* Are Encoded in the Wasp Genome

**DOI:** 10.1534/g3.119.400349

**Published:** 2019-11-01

**Authors:** Brian Wey, Mary Ellen Heavner, Kameron T. Wittmeyer, Thomas Briese, Keith R. Hopper, Shubha Govind

**Affiliations:** * Biology Department, The City College of New York, 160 Convent Avenue, New York, 10031,; † PhD Program in Biology, The Graduate Center of the City University of New York,; ‡ PhD Program in Biochemistry, The Graduate Center of the City University of New York, 365 Fifth Avenue, New York, 10016,; § Laboratory of Host-Pathogen Biology, Rockefeller University, 1230 York Ave, New York, 10065,; ** USDA-ARS, Beneficial Insect Introductions Research Unit, Newark, DE 19713, and; †† Center of Infection and Immunity, and Department of Epidemiology, Mailman School of Public Health, Columbia University, New York, 10032

**Keywords:** Extracellular vesicle, whole genome sequencing, *Leptopilina heterotoma*, endoparasitoid wasp, *Drosophila*, VLP, host-parasite, organelle, immune suppression

## Abstract

*Leptopilina heterotoma* are obligate parasitoid wasps that develop in the body of their *Drosophila* hosts. During oviposition, female wasps introduce venom into the larval hosts’ body cavity. The venom contains discrete, 300 nm-wide, mixed-strategy extracellular vesicles (MSEVs), until recently referred to as virus-like particles. While the crucial immune suppressive functions of *L. heterotoma* MSEVs have remained undisputed, their biotic nature and origin still remain controversial. In recent proteomics analyses of *L. heterotoma* MSEVs, we identified 161 proteins in three classes: conserved eukaryotic proteins, infection and immunity related proteins, and proteins without clear annotation. Here we report 246 additional proteins from the *L. heterotoma* MSEV proteome. An enrichment analysis of the entire proteome supports vesicular nature of these structures. Sequences for more than 90% of these proteins are present in the whole-body transcriptome. Sequencing and *de novo* assembly of the 460 Mb-sized *L. heterotoma* genome revealed 90% of MSEV proteins have coding regions within the genomic scaffolds. Altogether, these results explain the stable association of MSEVs with their wasps, and like other wasp structures, their vertical inheritance. While our results do not rule out a viral origin of MSEVs, they suggest that a similar strategy for co-opting cellular machinery for immune suppression may be shared by other wasps to gain advantage over their hosts. These results are relevant to our understanding of the evolution of figitid and related wasp species.

Parasitic wasps are among the most abundant insects; they are vital to biodiversity and contribute to biological control of agricultural pests (Narendran 2001; Rodriguez *et al.* 2013). A common strategy for reproductive success of parasitic wasps is suppression of immunity in their larval hosts. Parasitic wasps produce viruses or virus-like particles in tissues associated with the ovary. Wasps of the Ichneumonoidea superfamily produce symbiotic polydnaviruses (PDVs), which package circular dsDNA. PDV (Bracovirus (BV) in braconid wasps; Ichnovirus (IV) in ichneumonid wasps) genomes are integrated within the wasp genome as islands of viral genes. Upon oviposition, PDVs suppress host immunity. BVs and IVs derive from nudivirus and large DNA cytoplasmic viruses, respectively (reviewed in Strand and Burke 2015; Drezen *et al.* 2017; Gauthier *et al.* 2018, and references therein).

Immune-suppressive virus-like particles (VLPs) (*e.g.*, VcVLPs in the ichneumonid *Venturia canescens* and FaENVs in the braconid *Fopius arisanus*) lack proviral DNA segments, but are of viral origin and transfer virulence proteins into host cells (Pichon *et al.* 2015; Burke *et al.* 2018). Viral genes encoding VLP proteins are either dispersed in the wasp genome (as in VcVLP) or present in discrete genomic areas (as in FaENV). Thus, various independent viral endogenization events have been important for successful parasitism by these wasps (Strand and Burke 2015; Gauthier *et al.* 2018).

Here, we focus on immune-suppressive particles of figitid wasps in the genus *Leptopilina*, that infect *Drosophila* spp. and are gaining importance as models for natural host-parasite interactions (Keebaugh and Schlenke 2014). *Leptopilina heterotoma* (*Lh*), *L. victoriae* (*Lv*), and *L. boulardi* (*Lb*) produce VLPs in their venom glands. The VLPs of *Leptopilina* spp. and their proteins have been linked to parasite success (Rizki *et al.* 1990; Dupas *et al.* 1996; Morales *et al.* 2005; Labrosse *et al.* 2005; Chiu *et al.* 2006; Heavner *et al.* 2014). Evidence for DNA in *Leptopilina* VLPs is lacking, and because of the absence of a published wasp genome, the chromosomal *vs.* extrachromosomal location of MSEV protein genes is not known. Our goals here are (a) to describe additional proteins in the MSEV proteome and examine their relationship with PDV and other viral proteins, and (b) determine whether MSEV genes are encoded in the wasp genome.

We recently described 161 proteins in the VLPs from two *Lh* strains in three classes: conserved eukaryotic with cellular function (Class 1), infection- and immunity-related (Class 2), and unannotated (novel) without similarity to known proteins (Class 3) (Heavner *et al.* 2017). Class 1 proteins include several vesicular transport and endomembrane system proteins. Class 2 proteins include predicted modulators of immune response, *e.g.*, metalloendopeptidases, RhoGAPs, a knottin-like protein, and a new family of prokaryotic-like GTPases whose genes lack introns. A striking example of Class 3 proteins is p40, with three-dimensional structural similarity to Type 3 secretion system (T3SS) needle-tip proteins, IpaD/SipD/BipD from Gram-negative bacteria, *Salmonella*, *Shigella* and *Burkholderia*. Earlier results have indicated that the *p40* gene (unlike the *GTPase* genes) is expected to have introns. These results suggested that *Lh* VLPs have novel properties with elements of the prokaryotic and eukaryotic secretion systems and possess a functionally diverse array of immune-suppressive proteins. We therefore renamed VLPs as Mixed Strategy Extracellular Vesicles (MSEVs). Their variable morphologies distinguish them from ordered PDV morphologies. Additionally, genes encoding abundant MSEV proteins p40 and GTPase are present even in antibiotic-treated wasps. These results favored a non-microbial nature for MSEVs (Heavner *et al.* 2017).

Here, we present an analysis of an additional 246 proteins from the *Lh* 14 MSEV proteome to obtain a more comprehensive description. A combined analysis of these and previous results reinforce the idea that the MSEV proteome is enriched in exosomal proteins and that Class 3 proteins are not shared with either *Lb* or an unrelated *Ganaspis* spp. Whole-body transcriptome of adult *Lh* wasps validated the expression of the MSEV genes. *De novo* genomic assembly and analyses revealed 90% of conserved Insecta Benchmarking Universal Single-Copy Orthologs (BUSCOs), as well as a majority (375/407; ∼90%) of the MSEV proteins are encoded in the wasp genome. While we cannot rule out a viral origin of MSEVs, in aggregate, our results provide a clearer understanding of the current nature of these complex structures and strengthen the idea that specialized extracellular vesicles transfer wasp virulence factors and other parasite proteins into *Drosophila* host cells.

## Materials and Methods

### Insects

Isogenized *Lh* strains New York (NY; (Chiu and Govind 2002; Chiu *et al.* 2006)) and *Lh* 14 (Schlenke *et al.* 2007), were raised on the *y w* strain of *D. melanogaster* that were reared on standard cornmeal, yeast, and agar fly food at 25° as described by Small *et al*. (2012). Adult wasps were collected from parasitized hosts, 25 days after infection at 25°. Male and female wasps were stored on fly food with 70% honey on “buzz” plugs.

### Analysis of MSEV super-set ORFs

Previously undescribed open reading frames (ORFs) from the *Lh* 14 MSEV proteome and sequenced as part of Heavner *et al*. (2017) (PXD005632) are analyzed in the context of the published female abdominal (Goecks *et al.* 2013) and whole body (this study) *Lh* 14 transcriptomes. We have not observed any difference in venom activities of *Lh* 14 and *Lh* NY (Morales *et al.* 2005; Schlenke *et al.* 2007), or in wasp success under laboratory conditions. The *Lh* 14 ORFs were aligned against transcripts from BioProject: PRJNA202370, Accession number GAJC0000000 (Goecks *et al.* 2013) as previously described in Heavner *et al*. (2017). Proteins with an ORF and a transcript were run through the BLAST2GO (v 5.2; downloaded June 2018) annotation pipeline with an E-value threshold of 1x10^−7^ (Conesa *et al.* 2005; Götz *et al.* 2008). Results were organized and classified based on Gene Ontology (GO) terms from UniProt and InterPro (Ashburner *et al.* 2000; Jones *et al.* 2014; Uniport Consortium 2015; The Gene Ontology Consortium 2019). Proteins were considered “virulence-related” based on GO terms indicating involvement with infection, host evasion, inflammation, and immune response. ORFs that did not return results via BLAST or InterProScan (Class 3 proteins) were run through Conserved Domain Search (CDD) on NCBI (version 3.16) (Marchler-Bauer *et al.* 2017). The E-value cut off for CDD search was 1x10^−2^. Proteins were considered to have a signal peptide if one was predicted using Phobeus and Signal P (Käll *et al.* 2004; Käll *et al.* 2007; Nielsen 2017; Almagro Armenteros *et al.* 2019). Transmembrane domains were considered to be present if they were predicted using Phobeus and TMHMM (Sonnhammer *et al.* 1998; Krogh *et al.* 2001; Käll *et al.* 2004; Käll *et al.* 2007).

The GhostKOALA algorithm (Kanehisa *et al.* 2016) was used to assign KEGG ortholog (KO) numbers for the MSEV superset protein sequences. If a primary KO number failed to be assigned by GhostKOALA, a secondary number assignment with a score >= 50 was used. Redundant KO numbers were excluded.

MSEV proteins were included in the enrichment analyses only if a human ortholog exists; the gene identifiers for human orthologs were obtained from the MSEV KO and the UniProt mapping utility (Li *et al.* 2015; Uniport Consortium 2015). (Human orthologs were chosen because a robust proportion of Vesiclepedia’s data are derived from human vesicle proteomes.) The orthologs of human genes were analyzed for enrichment with the FunRich algorithm (Pathan *et al.* 2015; Pathan *et al.* 2017) against the Vesiclepedia database (Kalra *et al.* 2012; Pathan *et al.* 2019).

Finally, the MSEV proteome was used as a query using BLASTp for the following databases: “non-redundant” (nr), nr restricted to Taxid: Viridae (10239), nr restricted to Taxid: Polydnaviridae (10482), and nr restricted to Taxid: Unclassified Polydnaviridae (40273) (E-value threshold: 1.0x10^−3^, %ID minimum: 20%, performed 04/16/2019). tBLASTn of *L. boulardi* and *G. hookeri* (previously called *Ganaspis spp. 1*) (Goecks *et al.* 2013) transcriptomes was performed on 03/10/2019; the threshold for homologs in *Lb* and *G. hookeri* were 25% ID and an E-value of 1.0x10^−10^.

### Genomes sequencing and assembly

Library preparations, sequencing reactions, and associated validations were conducted by GENEWIZ, Inc. (South Plainfield, NJ, USA). Genomic DNA was extracted from ∼50 mg of tissue (∼100 wasps) of *Lh* males and females separately using mixed bead beating and PureLink Genomic DNA extraction kits following manufacturer’s protocol. Quantification of extracted DNA was performed using Nanodrop and Qubit2.0 Fluorometer (Live Technologies, Carlsbad, CA, USA). Integrity of genomic DNA was verified by gel electrophoresis (0.6% agarose). DNA libraries were prepared for each wasp gender by acoustic shearing fragmentation using a Covaris S220. Fragments were end repaired and adenylated prior to adapter ligation on 3′ ends (NEB NextUltra DNA Library Preparation kit, Illumina, San Diego, CA, USA). Enrichment and indexing of adapter-ligated DNA was done through limited cycle PCR. DNA library validation was performed using TapeStation (Agilent Technologies, Palo Alto, CA, USA). Libraries were quantified using Qubit 2.0 Fluorometer.

Real time PCR (Applied Biosystems, Carlsbad, CA, USA) was used to quantify DNA molar mass for each library before multiplexing in equal molar mass. DNA libraries were sequenced using a 2x150 paired-end (PE) configuration on one lane on an Illumina HiSeq 4000. Image analysis and base calling were performed using the HiSeq Control Software (HCS) on the HiSeq instrument.

The average size of inserts (without adaptors) in the Illumina library was ∼300-350 bp. *De novo* assembly of reads and scaffolding of contigs was performed using ABySS 2.2 (Jackman *et al.* 2017) by the New York Genome Center. *De novo* assembly of combined male/female genome was performed using Platanus-allee (Kajitani *et al.* 2019) and scaffolding was improved using AGOUTI (Zhang *et al.* 2016) on the University of Delaware’s BIOMIX cluster.

Sequences from *Drosophila*-associated bacteria such as *Wolbachia* spp., *Acetobacter pasteurianus*, and *Lactobacillus plantarum* were identified in both assemblies. *Wolbachia* are endosymbionts of many insects including *Leptopilina* spp. (Pannebakker *et al.* 2004; Werren *et al.* 2008; Gueguen *et al.* 2012). *Lactobacilli* and *Acetobacter* are symbionts and commensals of sugar-consuming insects (Crotti *et al.* 2010; Engel and Moran 2013). Among the three bacterial species, *Wolbachia* sequences were the most abundant. BLASTx analysis showed that predicted genes from *Wolbachia* scaffolds were associated with *Wolbachia* proteins in GenBank. These bacterial and mitochondrial sequence-containing scaffolds were identified during the NCBI submission process and were manually removed from the submission.

### Evaluation of genome assemblies

Assemblies made with ABySS and Platanus-allee with AGOUTI were run through QUAST v4.0 (Mikheenko *et al.* 2016) to determine scaffold number, N50, and GC%. All assemblies were examined for conserved genes and orthologs with BUSCO v9 (Simão *et al.* 2015; Waterhouse *et al.* 2017) using the Insecta set and training parameters set to “Nasonia”. NCBI BLAST+ (v 2.7.1) was used to compare selected scaffolds produced from male and female genome assemblies (Johnson *et al.* 2008; Camacho *et al.* 2009). E-value threshold was set at 1x10^−7^. E-values of alignments were considered acceptable if within the range of 0 to 1x10^−10^.

K-mer analysis was performed using the K-mer Analysis Toolkit (KAT) (Mapleson *et al.* 2016) and heat maps were used to compare multiplicity (coverage plus repeats) of K-mers to GC content of the reads, coloring bins according to the number of distinct K-mers in each. This analysis was used to determine whether there were separate clusters of multiplicity/GC content that might arise from different sources, such as contamination. BLAST (Altschul *et al.* 1990; Camacho *et al.* 2009; Johnson *et al.* 2008) was used to search for homologs of a random sample of genomic scaffolds to which reads from each cluster mapped. The joint assembly of the *Lh 14* genome was compared to the published *L. clavipes* genome (Bioproject: PRJNA84205 (Kraaijeveld *et al.* 2016)) through maps of 27-mer multiplicity *vs.* GC content. Finally, 27-mer multiplicity/GC content of the scaffolds (9.6 Mb) containing MSEV genes was compared to a random subset of scaffolds (9.6 Mb) without MSEV genes. Statistical differences between *Lh 14* and *L. clavipes* genomes and between MSEV-gene containing scaffolds and non-MSEV-gene containing scaffolds were calculated using a multivariate Cramér test (Ihaka and Gentleman 1996; Baringhaus and Franz 2004; Franz 2019).

### Gene predictions, gene annotation, and viral gene searches

Gene predictions were performed on parallel and anti-parallel strands using AUGUSTUS (v3.3.1; August 2018) (Stanke *et al.* 2004; Stanke and Morgenstern 2005; Keller *et al.* 2011) with the *Nasonia* training set. The AUGUSTUS readout was separated into mRNA, coding DNA sequence (CDS), and translations by gffread (Trapnell *et al.* 2012).

Gene predictions were annotated by performing a BLASTx of all gene predictions against the entire nr database (Downloaded on January 2019) and InterProScan on the University of Delaware BIOMIX Cluster before using BLAST2GO (Conesa *et al.* 2005; Götz *et al.* 2008) to finish annotation based on BLASTx and InterProScan results.

NCBI BLAST+ (v 2.7.1) was used on a local machine to search predictions and scaffolds, cutoff was %ID >70%, E-value < 1E-50, and query coverage > 70%. MSEV genes and 1x10^−2^ for Polydnavirus and Nudivirus proteins. Family *Polydnaviridae* and *Nudiviridae* protein sequences for the 11 species available on OrthoDB v9 were downloaded on February 2019 (Johnson *et al.* 2008; Camacho *et al.* 2009).

### Whole-body transcriptome sequencing and assembly

Total RNA extraction, library preparations, sequencing reactions, and bioinformatics analysis were conducted at GENEWIZ, INC (South Plainfield, NJ, USA). RNA was extracted from frozen tissue with the Qiagen RNeasy Plus Universal mini kit using manufacturer’s instructions (Qiagen, Hilden, Germany). The extracted RNA was quantified using a Qubit 2.0 Fluorometer and its integrity was checked with the 4200 TapeStation (Agilent Technologies, Palo Alto, CA, USA).

RNA samples were enriched for mRNA using Oligo d(T) beads. RNA sequencing libraries were prepared using the NEBNext Ultra RNA Library Prep Kit for Illumina following manufacturer’s instructions (NEB, Ipswich, MA, USA). The sequencing libraries were validated by using the Agilent TapeStation. Quantification was performed using the Qubit 2.0 Fluorometer and quantitative PCR (KAPA Biosystems, Wilimington, MA, USA).

Sequencing libraries were clustered on a single lane of a flow cell and sequenced on the Illumina HiSeq 4000 instrument using a 2x150 PE configuration. Image analysis and base calling were conducted by the HCS. Raw sequence data (.bcl files) was converted into fastq files and de-multiplexed using Illumina’s bcl2fastq 2.17 software. One mismatch was allowed for index sequence identification.

The Trinity v2.5 (Grabherr *et al.* 2011), *de novo* assembler was used to assemble the *Lh 14* transcripts. The *de novo* assembled transcriptome was created with a minimum contig length of 200 bp per sample. Transrate v1.0.3 (Smith-Unna *et al.* 2016) was used to generate statistics for the *de novo* assembled transcriptome. EMBOSS tools getorf were then used to find the ORFs within the *de novo* assembled transcriptome. The *de novo* transcriptome assembly was then annotated using Diamond BLASTx (Buchfink *et al.* 2015).

The transcriptome reads were mapped to the genomic scaffolds for downstream analyses using HISAT2 or BWA (Li and Durbin 2009; Kim *et al.* 2015).

### Preparation of template DNA and PCR

Male and female wasps (n = 12, for each sex), were separated and washed in 70% ethanol, and then rinsed twice in deionized water. Genomic DNA (gDNA) was extracted using a Qiagen DNeasy Blood and Tissue kit following provided protocols. gDNA was eluted in Tris-EDTA buffer, pH 8.0, and stored at 4°. The concentration of gDNA was determined by NanoDrop (Thermo Fisher).

For cDNA preparation, male and female wasps (n = 12 for each sex), were separated and washed in 70% ethanol and rinsed twice in deionized water. Total body RNA was extracted using 100 µL of Trizol (Invitrogen) following manufacturer’s protocols. RNA was resuspended in 0.1% DEPC treated water and treated with DNase I to remove contaminating DNA (Thermo Fisher Scientific). The RNA concentration was determined by NanoDrop (Thermo Fisher). cDNA was synthesized using Proto-Script First Strand cDNA Synthesis Kit (New England Biolabs).

### Analysis of select genes

Primers for *p40 and SmGTPase01* are as follows:

*p40* forward: GAATCATTGTTCGTTTGCTTGAAGAAAGAATTGG

*p40* reverse: CATTATTAATGGGCCTTTACAATAATTTTAGCC

*SmGTPase01* forward: CGTTGCACTACCTTGTTTGTCA

*SmGTPase01* reverse: TTGTCTTTGCCCTGAGCGTT

PCRs were performed with Taq polymerase (gift of C. Li lab, CCNY), PCR buffer (300 mM Tris HCl pH 9.5, 75 mM (NH_4_)_2_SO_4_, 10 mM MgCl_2_) and deoxyribonucleotides (0.2 mM; Thermo Fisher Scientific). The PCR products were resolved on a 1% agarose gel in Tris acetic acid EDTA buffer (40 mM Tris HCl pH 7.6, 20 mM acetic acid, 1mM EDTA pH 8.0). Ethidium bromide (Sigma Aldrich)-stained gels were visualized on an ultra violet Trans-Illuminator (UVP) and gel images were taken using the DigiDocIt Imaging System (UVP). Gel images were processed in Adobe Photoshop for clarity only.

gDNA or cDNA-containing PCR products were cloned into pCR TOPO II plasmids (Invitrogen) and transformed into DH10β competent cells (New England Biolabs). For plasmid preparation, colonies were screened via PCR and positive colonies were cultured in Luria Broth with ampicillin (100 µg/mL) at 37° overnight. Plasmids were extracted using Plasmid Miniprep kit (Qiagen) and sequenced (GENEWIZ, INC. South Plainfield, NJ, USA). Sequences were aligned using NCBI BLAST+ (Johnson *et al.* 2008; Camacho *et al.* 2009) and Clustal Omega (Li *et al.* 2015). Expected PCR band sizes were determined using SerialCloner (v2.6.1).

### Data availability

*L. heterotoma* strains (Chiu *et al.* 2006; Schlenke *et al.* 2007) are available upon request. File S1 contains details of supplemental files and tables. File S2 contains listing of accession numbers for all sequences reported in this work. Figure S1 contains the 27-mer *vs.* GC count comparison of MSEV containing scaffolds to non-MSEV containing scaffolds. Table S1 contains annotations and related data for proteins. Table S2 contains BLAST search results of the MSEV proteome against the nr database. Table S3 contains all BUSCOs found in male, female, and joint genome assemblies. MSEV protein sequences are available upon request. Accession numbers for datasets are as follows: *Leptopilina heterotoma* strain *Lh* 14, genome assembly: Male genome: QYUB0000000, Female genome: QYUC0000000, Joint genome: VOOK00000000. *Leptopilina heterotoma* strain *Lh* 14, whole-body transcriptome: GHUQ00000000. *Leptopilina heterotoma* abdominal transcriptome by Goecks *et al.*: GAJC0000000. *Leptopilina clavipes* genome Bioproject: PRJNA84205. *Leptopilina heterotoma* strain *Lh* 14 proteome: PRIDE: PXD005632. Supplemental material available at figshare: https://doi.org/10.25387/g3.10005260.

## Results and Discussion

### The MSEV proteome superset

**Figure 1:**
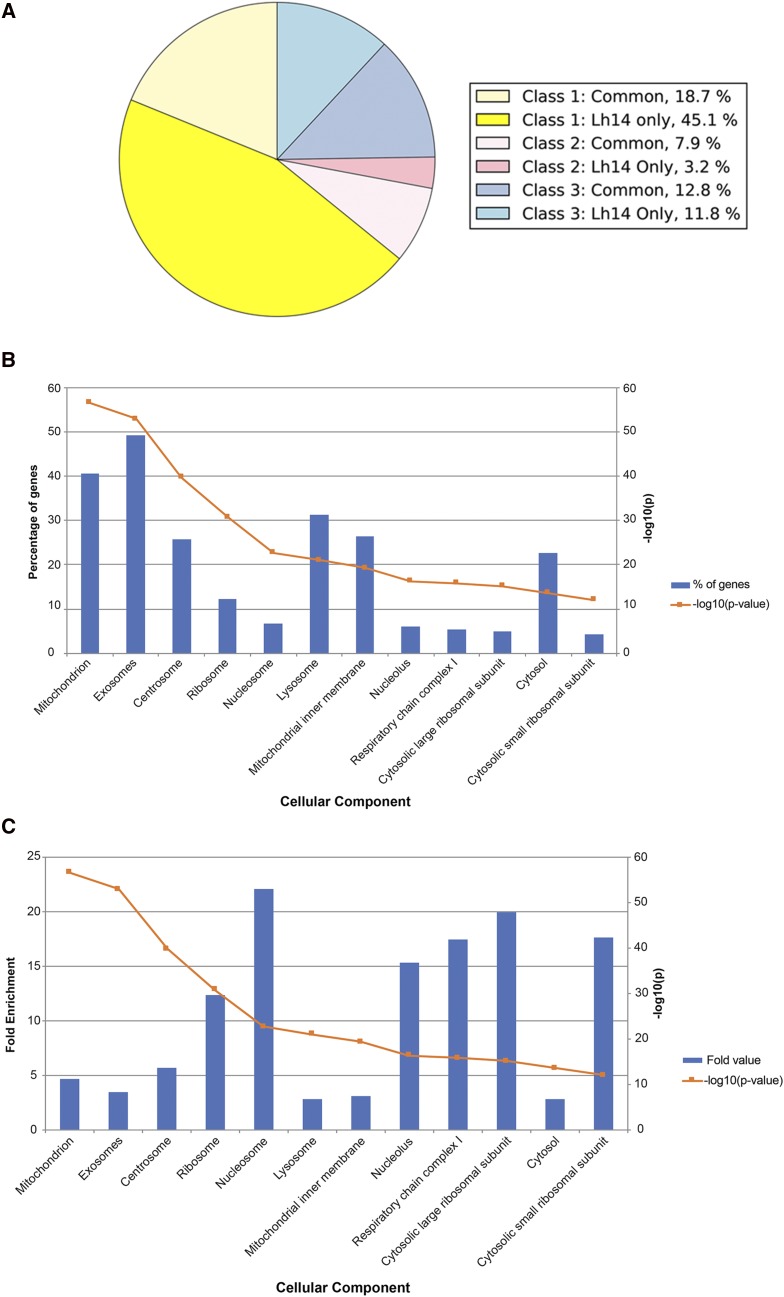
The superset of MSEV proteins: (A) *Lh* 14 MSEV proteins were annotated using BLAST2GO prior to class sorting via annotation and GO Terms. Wedges denoted as “Common,” were previously published in (Heavner *et al.* 2017) and represent proteins found in both *Lh* 14 and *Lh* NY MSEV proteomes. New proteins analyzed in this work are in wedges labeled “*Lh* 14 Only.” A majority of proteins belong to Class 1. Table S1 lists 246 proteins added to the superset *Lh* 14 proteome. (B) and (C) Enrichment analysis of MSEV superset shows high association with exosomes and mitochondria compared to other cellular organelles according to Vesiclepedia. –log _10_ (p-value) trend shown in orange for both graphs. The p-values were calculated with the Bonferroni method. (B) Percentage of MSEV genes associated with specific cellular compartments found in Vesiclepedia, relative to all MSEV genes. Of the superset proteins, 41 and 49% are associated with mitochondria and exosomes, respectively (*P* = 3 × 10^−57^; 1 × 10^−53^). (C) Fold-enrichment of the MSEV dataset in specific cellular compartments. Although many protein classes are present in the proteome, exosomal and mitochondrial proteins show more significant enrichments.

A comparative study of the proteomes of the MSEVs from *Lh* 14 and *Lh* NY strains previously generated a list of 161 “common” MSEV proteins (Heavner *et al.* 2017). More than 90% of the 161 proteins are part of the *Lh* 14 MSEV proteome. To describe MSEVs more completely, we characterized a larger set of 407 MSEV proteins from *Lh* 14 (161 common and 246 *Lh* 14) and define this set as the *Lh* MSEV “super-set” (Figure 1A). Key results from annotation-based classification, analysis for signal peptide and/or transmembrane domain, and presence/absence of proteins in related wasps are summarized below and in Table S1.

The presence/absence of signal peptide (SP) alone, or SP with/without the transmembrane (TM) domain(s) in MSEV proteins reveals their possible location (*i.e.*, potentially secreted into the venom gland lumen or associated with MSEV membrane). We therefore searched the 246 *Lh* 14 proteins for SP and TM domains. Of the 246 proteins, 55 (22.35%) have a predicted SP domain, 37 (15.04%) have a predicted TM domain, while 6 (2.44%) have both a predicted SP and TM domain.

After annotation, we found that a majority (183/246 or 75%) of the 246 proteins can be classified as core eukaryotic cell biology proteins (Class 1); 13/246 (5%) proteins as virulence- and immunity-related based on associated GO terms (Ashburner *et al.* 2000; The Gene Ontology Consortium 2019) (Class 2); and 50/246 or 20% as novel sequences without high confidence annotation (Class 3) (Table S1). A presence/absence analysis of these 246 proteins in published transcriptomes (Goecks *et al.* 2013) of *Lb* or a more distantly related wasp, *G. hookeri* (for thresholds see Methods) revealed the following: only 43/246 (17%) *Lh* MSEV proteins are expected to be found in *Lb* and/or *G. hookeri* (Table S1). Of these, 33/43 (77%) proteins were in Class 1 but only 7/43 (16%) and 3/43 (7%) were in Class 2 and Class 3 categories, respectively. These results support the idea that, multiple but different, infection strategies and/or host evasion strategies might exist among different wasps infecting the same hosts.

While most of the Class 1 proteins were annotated as ribosomal or mitochondrial-related, a few were described as integral membrane proteins, vesicle trafficking protein SEC22b (E-value: 6.22E-145), and the ion channels sideroflexin 1 and 2 (E-value: 0). We also identified an apolipophorin (E-value: 1.02E-7) (Table S1). The presence of these membrane-associated proteins reinforces the vesicular nature of MSEVs.

Examples of Class 2 proteins include the neural/ectodermal development factor IMP-L2 (E-value: 5.29x10^−50^) and a protein involved in pain reception, CG9231 (E-value: 4.39x10^−15^). A viral-like Diedel protein (E-value: 1.77x10^−7^), viral Enhancin (E-value: 6.02x10^−5^), l(2)37Cc (E-value: 3.39x10^−165^), odorant binding protein 56d-like (E-value: 5.64x10^−50^), major royal jelly protein (E-value: 8.59x10^−135^), and two venom acid-phosphatases Acph-1 (E-value: 4.12x10^−5^) were also found in the Class 2 category; their cDNA sequences were published previously (Heavner *et al.* 2013) (Table S1). It is possible that these MSEV proteins modulate the hosts’ immune responses and/or influence host development to facilitate successful parasitism.

Within Class 3, 45 proteins (90%) lacked BLASTp and InterProScan results. However, Conserved Domain Database (CDD) (Marchler-Bauer *et al.* 2017) searches returned 9 hits identifying potentially functional domains (Table 1). This included (a) a CD99L2 like antigen (%ID: 24%, E-value: 3x10^−3^), (b) a DEAD-like helicases superfamily member (%ID: 22%, E-value 2x10^−4^) and (c) a herpes outer envelope glycoprotein 350 (gp350), (%ID: 28%, E-value: 4x10^−3^) (Table 1).

**Table 1:** CDD-search results of MSEV “un-annotated” proteins in the super-set. MSEV ORFs that completed the BLAST2GO pipeline and did not return any results were run through the NCBI CDD-Search Version 3.16 (Accessed: Aug. 2018). Of 45 queries, only 9 returned hits with threshold set to 1x10^−2^. The ninth result came from a search with E-value threshold set to 1. Results listed are all unique, high scoring hits for each ORF that returned hits from the search

MSEV Superset Unknowns CDD-Search Results						
Query (in-house ID)	PSSM-ID	From	To	E-Value	Accession	Short name
GAJC01013214.1_14	**331760**	**25**	**98**	**0.000176**	cl26939	DEXDc superfamily
GAJC01012558.1_12	**330317**	**39**	**205**	**0.003987**	cl25496	Herpes_BLLF1 superfamily/gp350
GAJC01011863.1_13	**311912**	**86**	**187**	**0.003653**	cl07006	RNA_polI_A34 superfamily
GAJC01011463.1_48	**315064**	**234**	**335**	**0.002964**	cl13702	CD99L2 superfamily
GAJC01010930.1_16	**328726**	**32**	**61**	**0.001252**	cl21457	ICL_KPHMT superfamily
GAJC01010353.1_14	**331876**	**31**	**121**	**0.001483**	cl27055	MutS_III superfamily
GAJC01009713.1_25	**311628**	**138**	**225**	**0.000133**	cl06688	TSGP1 superfamily
GAJC01009493.1_4	**328724**	**79**	**96**	**0.001983**	cl21455	P-loop_NTPase superfamily
GAJC01002124.1_43	**330572**	**4**	**269**	**0.0073146**	cl25751	DUF4045 superfamily

A BLASTp DELTA-BLAST of the potential gp350 domain against the nr database specifying “Vira” (taxid: 10239) under organism resulted in Crimean-Congo hemorrhagic fever orthonairovirus envelope glycoprotein (%ID: 30%, E-value: 2.5x10^−1^), Lymphocryptovirus Macaca gp350 (%ID: 29%, E-value: 7.2x10^−1^), and Gallid Alphaherpesvirus 1 envelope glycoprotein J (%ID: 26%, E-value: 1.2) as top hits. BLASTp DELTA-BLAST of the potential gp350 domain against the nr database for Hymenoptera yielded an uncharacterized protein as the best hit (%ID: 24%, E-value: 8x10^−6^) in the ant *Vollenhovia emeryi*. This ant protein is predicted to contain calcium-binding EGF domains. The second hit in this search is from *N. vitripennis* for a predicted mucin-3A like glycoprotein (Gendler and Spicer 1995) (%ID: 24%, E-value: 2x10^−4^). Interestingly, transcripts related to the potential *Lh* gp350-like protein are not found in the *Lb* or *G. hookeri* transcriptomes (Table S1) (Goecks *et al.* 2013). Presence of this gp350-like protein in *Lh* MSEVs, but its absence in *Lb* MSEVs, suggests that it somehow contributes to differences in *Lh*/*Lb* host-parasite interactions and is therefore worthy of future studies. Complement receptor type 2 (CR2) in human B lymphocytes interacts with gp350 during Epstein-Barr infection (Young *et al.* 2008) and finding a verified homolog of CR2 in *Drosophila* hosts would be interesting in future research.

Because more than 200 proteins have now been added to the previously described MSEV proteome (Heavner *et al.* 2017), we re-evaluated our previous enrichment analysis. In an ortholog-based comparison of the superset to human extracellular vesicle (EV) proteomes in Vesiclepedia (the most current and robust source of EV data (Kalra *et al.* 2012)), we found that the largest proportion of superset proteins (49%) are proteins specifically associated with exosomes (Figure 1B). In human and mouse EV proteomes, mitochondrial and ribosomal proteins are enriched (Kalra *et al.* 2012). Accordingly, protein components of mitochondria (*e.g.*, respiratory chain) and ribosomes (*e.g.*, large and small subunit proteins) are found to be highly enriched in the *Lh* MSEV superset. However, we found that the significance of the enrichment was higher between the superset and exosomal proteins than mitochondrial or ribosomal proteins (Figure 1C). These results demonstrate the similarities in the protein profiles of MSEVs and EVs.

### Do Lh MSEVs contain homologs of PDV or viral proteins?

Even though figitid *Leptopilina* wasps are distantly related to PDV-containing Ichneumonid and Braconid wasps (Misof *et al.* 2014; Strand and Burke 2015), an association of PDV-like viruses in figitid wasps cannot be discounted because of shared evolutionary history. Recent publications have identified capsid-less VLPs in Ichneumonidae wasps (Volkoff *et al.* 2010; Pichon *et al.* 2015; Burke *et al.* 2018) and it is possible that *Lh* MSEVs have a similar viral origin. We therefore analyzed the *Lh* MSEV proteome superset against the GenBank PDV database, and then against its entire Viridae database.

To identify false positives, MSEV proteins with positive PDV hits (E-values were less than 1.0x10^−3^, %ID was 20% or greater, and query coverage was 30% or higher) were also searched against the unrestricted nr database to compare relatedness. If an MSEV protein is similar to a viral or virus-related PDV protein, we expected that, in the unrestricted nr database search, the MSEV query sequence would align again with the same viral subject sequences, but with a lower E-value (Table S2).

For PDV searches (Taxid: 10482 and Taxid: 40273), four proteins returned hits with E-values better than 1.0x10^−20^ and query coverage greater than 30%. Three of these hits are conserved proteins (cytochrome P450 and histone 4) while the fourth result identified an uncharacterized *Cotesia congregata* bracovirus (CcBV) protein (%ID: 31.08%, E-value: 1.38x10^−17^, query coverage: 77%) (Table S2). The unbiased BLASTp search against the entire nr database however had better results against eukaryotic proteins (E values: 0 to 2.0x10^−7^ and %ID from 100 to 56.25) (Table S2). In fact, the query that yielded the CcBV protein was better matched to a eukaryotic ribonuclease (%ID: 26.06%, E-value 1.14x10^−16^, query coverage: 84%) (Table S2). These results suggest that MSEV sequence similarities with PDV proteins may not be significant.

We also searched the *Lh* MSEV superset for presence of *L. boulardi* Filamentous Virus (LbFV) homologs (LbFV is a behavior manipulating virus of *Lb* (Varaldi *et al.* 2006; Patot *et al.* 2012)). Of LbFV’s 108 genes, 13 are present in genomes of *Lb*, *Lh* and related species, and the 13 transcripts are expressed in the *Lb* venom gland (Di Giovanni *et al.* 2019). Within our thresholds, we obtained only three (of 13) sequences with similarity to LbFV ORFs. However, these three *Lh* MSEV proteins, with hits for LbFV sequences obtained better scoring hits in the unrestricted nr database, suggesting that the *Lh* MSEV proteins are not highly related to the LbFV proteins (Table S2).

When comparing MSEV proteins to the entirety of Viridae, a total of 35 MSEV proteins had hits with %IDs ranging from 30 to 71% and E-values ranging from 1.0x10^−22^ to 1.0x10^−178^ (Table S2). However, a BLASTp search against the entire nr database found that proteins with results for viral hits had better scores when searched against the entire nr database, indicating that while viral hits are possible, they are not the best match (Table S2). This result, in addition to the fact that 372 other MSEV proteins (including the Diedel and Enhancin (Table S1)) did not return viral hits, would indicate that a majority of MSEV proteins are not closely related to viral proteins.

### The whole-body transcriptome contains expressed MSEV transcripts

We performed a mixed-gender whole-body transcriptome sequencing and *de novo* assembly of *Lh* transcripts. This assembly generated 104,066 transcripts. This dataset is more than three times larger than the published data derived from abdomens of female wasps that has 31,400 transcripts (Goecks *et al.* 2013). A BLAST analysis of the female abdominal transcripts against the male/female whole-body transcripts showed that a majority (21,493/31,400, 68.4%) were present in the latter data set.

We searched the whole-body transcriptome for MSEV protein coding sequences using tBLASTn. Of 407 MSEV superset proteins, we identified transcripts for 371 (91.1%) proteins. Despite the ∼9% discrepancy (likely due to differences in expression levels due to different experimental conditions), these results largely verify the transcript data from (Goecks *et al.* 2013) that we have based our proteomic analyses on. Of the 371 MSEV transcripts identified, 233 (63%) encode Class 1, 44 (12%) encode Class 2, and 94/371 (25%) encode Class 3 proteins.

### Assembly of the Lh genome

We separately sequenced *Lh* 14 male and female genomic DNA and assembled the paired-end reads *de novo*, using ABySS (Jackman *et al.* 2017). These assemblies have a modest scaffold N50 of 4,800 with more than 100,000 scaffolds and an average coverage of 87% (Table 2). Assembly with MaSurCa (Zimin *et al.* 2013) provided similar results (data not shown), indicating that our assembly quality is limited likely due to factors such as large genome size and repetitive sequence regions (Dominguez Del Angel *et al.* 2018). Although the N50 values and large number of scaffolds indicate that the genome is not highly assembled, we found at least 80% of BUSCOs shared in the Insecta set in both assemblies (Table 2, BUSCOs in Table S3).

**Table 2:** Assembly statistics: Statistics of male, female, and combined (male plus female) *Lh* genomes as assessed by QUASTv4.0 and BUSCOv9.0. Percent coverage was found by mapping sequencing reads back to assembly using HISAT2. The identified BUSCOs can be found in Table S1. The QUAST program was run with parameters set for eukaryotic genomes and scaffolds. The BUSCO program was run with species set to ‘Nasonia.’ Contigs smaller than 500 bp were excluded

ASSEMBLY STATISTICS				
		Male	Female	Joint
Assembly	N50 (bp)	**4,779**	**4,843**	**11906**
	No. scaffolds	**147,558**	**147,549**	**83,487**
	Largest scaffold (bp)	**306,667**	**176,371**	**375,275**
	Total length (bp)	**474,383,205**	**472,302,230**	**462,564,754**
	GC%	**27.54**	**27.28**	**27.84**
	Coverage (%)	**87.7**	**86.8**	**91.1**
BUSCOs (Insecta)	Complete	**80.2%**	**81.3%**	**90.9%**
	Single	**69.4%**	**71.8%**	**89.2%**
	Duplicated	**10.8%**	**9.5%**	**1.7%**
	Fragmented	**15.9%**	**15.1%**	**6.5%**
	Missing	**3.9%**	**3.6%**	**2.6%**
	n	**1658**	**1658**	**1658**

While still fragmented, a *de novo* joint assembly of male and female sequences using Platanus-allee and AGOUTI improved assembly and scaffolding statistics (N50: 11,906, average coverage 91%). The number of found BUSCOs in the joint assembly rose to 90% (Table 2, BUSCOs in Table S3).

**Figure 2:**
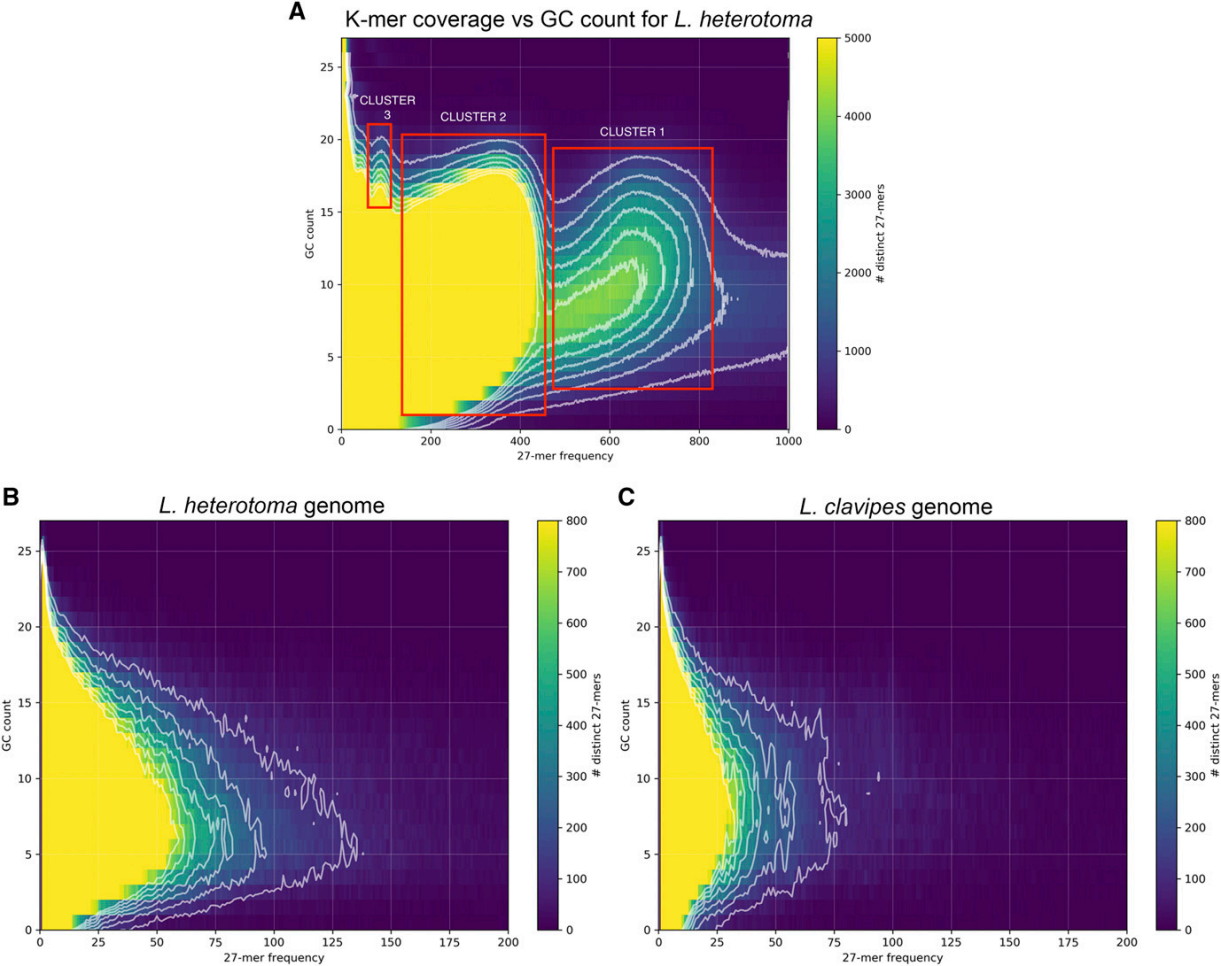
Analysis of K-mer coverage *vs.* GC count. (A) Analysis of genomic reads. 27-mers generated from the cleaned Illumina reads used to assemble the *L. heterotoma* genome binned by their GC count *vs.* multiplicity (total counts among the reads). Bins are colored by the number of distinct K-mers. Different clusters are identified as shown and described in the text. (B and C): A map of 27-mer multiplicity *vs.* GC content of the joint assembly of the *Lh 14* genome (B) to a map from the published *L. clavipes* genome (Bioproject: PRJNA84205) (C).

Analysis of K-mer multiplicity *vs.* GC content in the genome sequencing reads using the K-mer analysis tool, KAT (Mapleson *et al.* 2016) showed three possible clusters, although they are difficult to distinguish (Figure 2A). Cluster 1 has high multiplicity (450-650), Cluster 2 has lower multiplicity and a wide range of GC content, and Cluster 3 has the lowest multiplicity and the highest GC content. Cluster 3 overlaps with Cluster 2 making them hard to fully separate. BLAST searches of a random sample (1,672/4,482) from Cluster 1 contigs hit insect homologs 73% (1,220/1,672) of the time, *Acetobacter* homologs 13% (216/1,672) of the time, and then a variety of mostly Eukaryotic hits. Cluster 2 represents a majority of the wasp genome (>94%), and blast hits of a random sample (316/68,173) of its contigs almost exclusively had homologs in Hymenoptera (311/316; 98%) and mostly in *L. heterotoma* (227/316; 71%). Cluster 3 is the smallest of the three and contigs from Cluster 3 had homologs exclusively in *Acetobacter* (110/110). There was no evidence for contamination from a viral source or discrete MSEV-specific set of nucleic acid sequence.

Furthermore, K-mer multiplicity *vs.* GC content for the joint assembly of the *Lh 14* genome (Figure 2B) showed a very similar heat map to that using the published assembly of *L. clavipes* (Figure 2C; Bioproject: PRJNA84205 (Kraaijeveld *et al.* 2016)). The two genomes have highly similar 27-mer/GC profiles that do not differ statistically (multivariate Cramér test statistic = 114,119, *P* = 0.73, number bootstrap-replicates = 1000). The *L. heterotoma* assembly has 27-mers with approximately twice the multiplicity of those found in *L. clavipes*, which may represent increased repeat content in *L. heterotoma* and is supported by an assembly size over 200 Mb larger than the *L. clavipes* genome (463 Mb *vs.* 255 Mb) (Kraaijeveld *et al.* 2016).

### MSEV genes are encoded in the wasp genome

Using our annotation pipeline, 28,481 predicted genes were annotated. Within the annotated genes, we found 8 genes for the body color *yellow*, 3 *major royal jelly protein* (*mrjp*) genes, 25 odorant receptor/odorant binding protein coding genes, and 94 gene predictions for *cytochrome P450*. Some of these nuclear genes are not only involved in development and cellular processes, but are also included in the MSEV proteome (Table S1 and (Heavner *et al.* 2017)). A search of gene predictions for MSEV proteins via tBLASTn identified 325 of 407 (80%) MSEV sequences (Table 3). Of these, 153/407 (38%) had a percent identity of 95% or greater. Presence or removal of scaffolds with bacterial DNA sequences from either the separate male/female or the joint assembly did not affect this number, supporting the nuclear location of a majority of the MSEV genes.

**Table 3:** MSEV genes found in scaffolds and predictions: Gene predictions from genome assembly scaffolds and AUGUSTUS gene predictions were searched for MSEV genes using tBLASTn. Results better than %ID >70%, E-value < 1x10^−50^, and query coverage > 70% were retained

MSEV GENES FOUND IN GENOME ANALYSIS				
	MSEV BLASTn scaffold results		AUGUSTUS prediction results	
	Found	Percentage	Found	Percentage
Female	**278**	**68.3**	**169**	**41.5**
Male	**275**	**67.6**	**166**	**40.8**
Shared in M+F	**265**		**159**	
Joint Assembly	**375**	**92.1%**	**325**	**79.9%**

As gene prediction software can potentially miss genes (Wang *et al.* 2004), we searched the genomic scaffolds directly for MSEV-coding sequence regions using known protein sequences as queries via tBLASTn before and after removal of bacterial sequences. In both cases, 375/407 (92%) MSEV sequences were at least 70% complete as determined by query coverage (Table 3). Of these, 191/407 (47%) had a percent identity of 95% or greater.

The scaffolds containing MSEV genes (Fig. S1A) were also compared to a random subset of scaffolds without MSEV genes (Fig S1B) for their 27-mer/GC profiles. These appeared to not differ statistically (multivariate Cramér test statistic = 3755, *P* = 0.80, number bootstrap-replicates = 1000), indicating that the MSEV genes lie on scaffolds that resemble the rest of the genome.

### Characterization of select MSEV genes

We spot checked small portions of the genome for gene structure predictions of MSEV virulence protein genes *SmGTPse01* (Class 2) and *p40* (Class 3). For this, we sequenced PCR products of gDNA corresponding to these genes.

**Figure 3:**
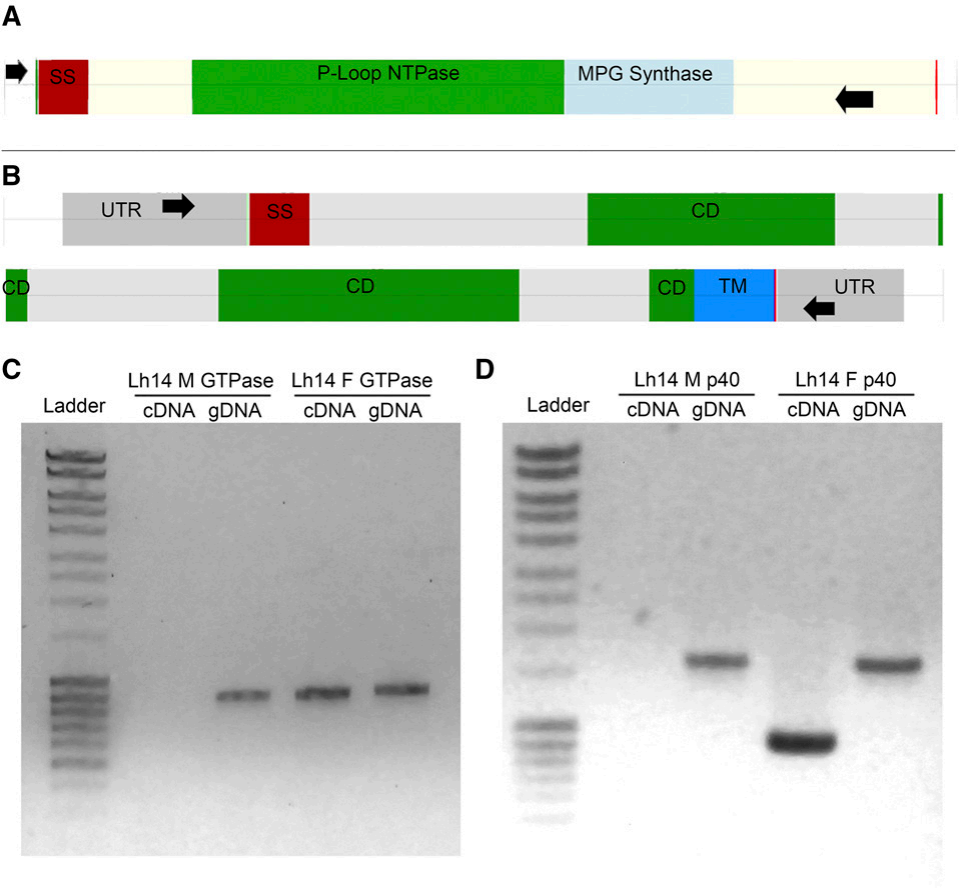
Predicted gene structures verified by PCR amplification experiments (A, B). Diagrams showing primer locations and predicted gene structures of *SmGTPase01* (A) and *p40* (B). Black arrows indicate primer locations, light gray indicates introns, UTR regions are dark gray and labeled, exons encoding potential protein domains are labeled as shown. Cream colored regions in panel A do not have a specified domain. Diagrams were drawn using GenomeDiagram as part of the Biopython (v. 1.6) package (Pritchard *et al.* 2006; Cock *et al.* 2009). Each row in the panels A and B diagrams corresponds to approximately 1,000 bp. For primer sequences, see methods. (C and D) Ladder is Thermo Fisher MassRuler ladder. (C) PCR products for *SmGTPase01* from male or female cDNA and gDNA. All products are 873 bp long. Male cDNA PCR was negative. (D) PCR products for *p40* from male or female cDNA and gDNA. The expected band for *p40* cDNA is 939 bp and for gDNA is 1,630 bp. Male cDNA PCR was negative. Sequence analysis of PCR amplification products confirmed gene prediction results.

The MSEV SmGTPase01 has prokaryotic-like GTPase domains and its gene is expected to lack introns (Heavner *et al.* 2017). The predicted *SmGTPase01* CDS spans 936 bp, which contains the functional GTPase domain (Heavner 2018). Scaffolds from male and female genomes confirmed the absence of coding region introns (data not shown). We hypothesized that primers in 5′ and 3′ untranslated regions (UTRs) should amplify the exact fragment from cDNA/gDNA as template based on manual characterization of the *SmGTPase01* locus (Heavner 2018) (Figure 3A). This prediction was borne out and we amplified an 873 bp fragment only from female cDNA and from both male and female gDNA (Figure 3C). The sequenced PCR products were identical to corresponding sequence within the assembly and the published transcript sequence from Goecks *et al.* (data not shown).

The *p40* gene encodes a protein that is structurally similar to T3SS bacterial needle-tip proteins IpaD/SipD from *Shigella* and *Salmonella*. However, *p40*’s genomic sequence is expected to have introns (Heavner *et al.* 2017). The full p40 gene was computationally assembled and predicted within both male and female genomes. Primers designed for *p40*’s 5′ and 3′ UTRs (Goecks *et al.* 2013; Ramroop 2016) (Figure 3B), allowed amplification of *p40*’s 939 bp cDNA only in preparations from female wasp extracts, but gDNA bands at 1,630 bp were detected from reactions when either male or female genome was used as template, indicating the presence of introns (Figure 3D). Sequencing the cloned cDNA product from females confirmed the published cDNA sequence (Heavner *et al.*, 2017). We also cloned and sequenced the gDNA products from male and female wasps and found the sequences to be identical (data not shown).

Unlike the well-characterized *Drosophila* hosts, the biology and molecular-genetics of their parasitic wasps have remained relatively obscure with only recent characterizations of *Leptopilina* and *Ganaspis* spp. (Melk and Govind 1999; Colinet *et al.* 2013; Goecks *et al.* 2013; Heavner *et al.* 2013; Mortimer *et al.* 2013; Heavner *et al.* 2017; Di Giovanni *et al.* 2019). Our proteomic, transcriptomic and genomic results here expand the available information on *L. heterotoma*. Bioinformatics analysis of the additional MSEV proteins does not alter the initial interpretation of the original 161 proteins. Genomic sequencing and analysis of scaffolds reveals that more than 92% of the MSEV genes reside on the wasp genome. We did not find evidence for MSEV gene association with endosymbiont or commensal bacterial DNA. We suspect that the remaining ∼8% are also nuclear genes and this association will be confirmed in higher quality assemblies. Altogether, these results strongly suggest that, like other subcellular structures, MSEVs are encoded in the wasp nuclear genome.

The cellular nature of *Lh* vesicles is likely to be shared by closely related *Lv* and *Lb* wasps. Our previous work has shown that the overall morphologies of *Lh* and *Lv* MSEVs are similar (Morales *et al.* 2005; Chiu *et al.* 2006). However, this is not the case for *Lb* MSEVs; different *Lb* strains have varying MSEV morphologies (Dupas *et al.* 1996; Gueguen *et al.* 2011; Wan *et al.* 2019). Interpretation of their identity also varies. For example, Di Giovanni *et al.* (2019) contend that MSEVs/VLPs are derived from a virus ancestral to the LbFV. Our analysis of the expanded proteomic superset does not lend strong support to this line of thinking.

We did not find convincing evidence of PDV or other viral structural proteins in the *Lh* MSEV proteome. However, we cannot discount that MSEVs have a viral origin as our analysis is limited by the fragmentation of the genome. It is also possible that a virus related to MSEVs may not have been identified to date. Mechanistically, eukaryotic viruses and vesicles share cellular pathways involving the endomembrane systems of their cells of origin or their target cells (Nolte-‘t Hoen *et al.* 2016), leading to overlap in protein functionality, but not necessarily origin. Thus, at least some of the Class 1 proteins in the MSEV proteome may be central to MSEV biogenesis in the wasp or for their interactions with the host hemocytes’ endomembrane machinery despite potentially being related to viruses. It is noteworthy that energy metabolism genes appear to be involved in rapid speciation and adaptation to new environments (Gershoni *et al.* 2009; Lane 2009), raising the possibility that MSEV mitochondrial proteins might contribute to this process. How *Lh* MSEVs are functionally similar to other insect or mammalian EVs remains to be explored experimentally. Functional characterization of predicted infection and immunity Class 2 proteins should explain the immune-suppressive strategies of these wasps. RNA interference, infection assays, and other experimental strategies should make this line of inquiry feasible.

Functional assignments are difficult for the unannotated Class 3 proteins. These are likely to be quite interesting, due to their different expression profiles in *Lh* *vs.* *Lb* and *G. hookeri* species. This difference in expression may stem either from *cis* changes in their regulatory sequences, or from absence of these genes in the *Lb* or *G. hookeri* genomes. Recent comparative genomics analysis has shown that over 40% of venom genes in the closely-related species *N. vitripennis* and *N. giraulti* have diverged significantly and up to 25% of venom genes are specific to a species (Martinson *et al.* 2017). A proteomic analysis of the venom genes of *Leptopilina spp*. and a molecular understanding of their expression will provide insights into how key activities within MSEVs evolved to parasitize the range of fruit fly hosts.

A key question regarding *Lh* virulence proteins critical to wasp success is whether their genes reside in a discrete region of the genome like a “virulence island” found in some microbial genomes (Dobrindt *et al.* 2004; Gal-Mor and Finlay 2006), or whether some genes are dispersed within the genome, while others occur in one or more clusters as in wasps with PDVs (Volkoff *et al.* 2010; Pichon *et al.* 2015). More complete assemblies, scaffolded to the level of chromosomes, will describe the genome-wide distribution of these genes in *Lh* and related wasps. Key MSEV genes could serve as genetic markers in future studies. Comparative genomics will uncover additional gene family members of MSEV proteins in other *Leptopilina* wasps and enable the development of new functional genomics tools such as CRISPR-disrupted mutant alleles made in *N. vitripennis* (Werren *et al.* 2009; Siebert *et al.* 2015; Li *et al.* 2017b; Li *et al.* 2017a). These approaches will open new avenues for understanding the biology of this host-parasite model.
